# Hyperhomocysteinemia exacerbates ischemia-reperfusion injury-induced acute kidney injury by mediating oxidative stress, DNA damage, JNK pathway, and apoptosis

**DOI:** 10.1515/biol-2021-0054

**Published:** 2021-05-29

**Authors:** Mei Zhang, Jing Yuan, Rong Dong, Jingjing Da, Qian Li, Ying Hu, Fangfang Yu, Yan Ran, Yan Zha, Yanjun Long

**Affiliations:** Department of Biomedicine, Guizhou University School of Medicine, Guizhou University, Guiyang 550025, Guizhou, People’s Republic of China; Division of Nephrology, Guizhou Provincial People’s Hospital, Guizhou Provincial Institute of Nephritic & Urinary Disease, No. 83 East ZhongShan Road, Guiyang 550002, Guizhou, People’s Republic of China

**Keywords:** hyperhomocysteinemia, ischemia-reperfusion injury, acute kidney injury, oxidative stress, JNK signaling pathway

## Abstract

**Background:**

Hyperhomocysteinemia (HHcy) plays an important role in the progression of many kidney diseases; however, the relationship between HHcy and ischemia-reperfusion injury (IRI)-induced acute kidney injury (IRI-induced AKI) is far from clear. In this study, we try to investigate the effect and possible mechanisms of HHcy on IRI-induced AKI.

**Methods:**

Twenty C57/BL6 mice were reared with a regular diet or high methionine diet for 2 weeks (to generate HHcy mice); after that, mice were subgrouped to receive sham operation or ischemia-reperfusion surgery. Twenty four hour after reperfusion, serum creatinine, blood urea nitrogen, and Malondialdehyde (MDA) were measured. H&E staining for tubular injury, western blot for γH2AX, JNK, p-JNK, and cleaved caspase 3, and TUNEL assay for tubular cell apoptosis were also performed.

**Results:**

Our results showed that HHcy did not influence the renal function and histological structure, as well as the levels of MDA, γH2AX, JNK, p-JNK, and tubular cell apoptosis in control mice. However, in IRI-induced AKI mice, HHcy caused severer renal dysfunction and tubular injury, higher levels of oxidative stress, DNA damage, JNK pathway activation, and tubular cell apoptosis.

**Conclusion:**

Our results demonstrated that HHcy could exacerbate IRI-induced AKI, which may be achieved through promoting oxidative stress, DNA damage, JNK pathway activation, and consequent apoptosis.

## 1 Introduction

Acute kidney injury (AKI) is a multiphasic clinical syndrome characterized by a rapid decline in renal function. Many factors such as ischemia/reperfusion [1], sepsis [2], trauma [3], and contrast [4] can induce the development of AKI. AKI is a common problem affecting hospitalized patients, with 25–40% mortality rates in severe cases [5]. A multicenter retrospective cohort study of 659,945 hospitalized adults from a wide range of clinical settings in nine regional central hospitals across China has reported that the incidence of community-acquired AKI and hospital-acquired AKI was 2.5 and 9.1%, respectively, giving rise to an overall incidence of 11.6% [6]. AKI is associated with poor clinical outcomes and long-term health and economic consequences; therefore, research about AKI has always been one of the focuses in kidney disease.

Homocysteine (Hcy) is an intermediate product of methionine metabolism. Hyperhomocysteinemia (HHcy), defined as blood Hcy concentration >15 µmol/L, is mainly developed by dysfunction of enzymes and cofactors associated with the biosynthesis and metabolism of Hcy. Other factors such as excessive methionine intake and certain diseases can also induce the development of HHcy [7]. Evidence has demonstrated that HHcy not only has a close relationship with the development of atherosclerosis, congestive heart failure, age-related macular degeneration, Alzheimer’s disease, and cancers, but also plays important roles in the progression of many kidney diseases such as chronic kidney disease (CKD) and diabetic nephropathy (DN) [7]. For instance, in a clinical study performed by Kong et al., the authors found HHcy increases CKD risk in a middle-aged and elderly Chinese population [8]. Xu et al. found serum Hcy was significantly higher in DN patients than simple diabetic patients and concluded that serum Hcy might serve as a biomarker for DN progression [9].

Except for CKD and DN, recent evidence also indicated that HHcy has a close relationship with AKI. For instance, Prathapasinghe and colleagues found that Hcy levels were significantly elevated after ischemia-reperfusion and neutralization of Hcy with anti-Hcy antibodies not only abolished ischemia-reperfusion-induced oxidative stress and cell death, but also transiently restored renal function [10]. In our previous studies, we have demonstrated that HHcy can exacerbate Cisplatin-induced AKI [11] and accelerate AKI to CKD progression by downregulating heme oxygenase-1 expression [12]; however, the relationship between HHcy and AKI, especially ischemia-reperfusion injury (IRI)-induced AKI, is far from clear.

In the present study, we used a high methionine diet (containing 2% methionine) to feed mice for 2 weeks to generate HHcy mice. After that, the IRI-induced AKI model was established to determine whether preexisted HHcy condition can exacerbate IRI-induced AKI through mediating oxidative stress, DNA damage, c-Jun N-terminal kinase (JNK) pathway, and apoptosis.

## 2 Materials and methods

### 2.1 Animals experiment

Twenty 6–7 weeks male C57BL/6 mice weighing 18.0–19.2 g were purchased from Liaoning Changsheng Biotechnology Co., Ltd (Benxi, Liaoning, China). Mice were bred and maintained in the Guizhou Medical University (Guiyang, Guizhou, China). All mice were reared under the temperature of 22 ± 2°C with a humidity of 55 ± 2% and a 12/12 h light cycle. After 1 week of habituation, all mice were randomly divided into two groups: the control diet group (*n* = 10) and the high methionine diet group (H-Met diet, *n* = 10). Diets were provided *ad libitum*. Two weeks after grouping, blood was collected through the caudal vein, and serum Hcy level was measured by a Hcy Assay Kit (Ausa, Shenzhen, China) and point-of-care testing device provided by the Shenzhen AoSA Company (Shenzhen, China). After that, the animals of each group were randomly divided into two groups again, including the control group and IRI group (*n* = 5). Mice in the IRI group were anesthetized with 1.5% pentobarbital sodium (45 mg/kg, i.p.) and placed on a homeothermic station to maintain body temperature at 37.5°C. The kidneys were exposed through bilateral incision and the renal pedicles were clamped for 30 min; the clamps were then released for reperfusion. After surgery, one milliliter of warm saline (37.5°C) was intraperitoneally injected for the purpose of volume supplement. Identical procedures except for clamping of the renal pedicle were done in the mice of the control group. Twenty four hour after reperfusion, blood samples were collected from the eyeball to test serum creatinine and blood urea nitrogen (BUN). Kidney tissues were fixed in 10% neutral-buffered formalin or snap-frozen for later use.


**Ethical approval:** The research related to animal use has been complied with all the relevant national regulations and institutional policies for the care and use of animals and was approved by the ethics committee of Guizhou Provincial People’s Hospital (ethics approval number: 2017057).

### 2.2 Evaluation of renal function

Serum creatinine and BUN, determined by creatinine and BUN assay kits purchased from the Bioassay system (USA), were used according to the manufacturer’s instructions to evaluate the renal function of animals.

### 2.3 Measurement of lipid peroxidation

Snap-frozen tissues were used to determine the lipid peroxidation levels in the kidney by measuring malondialdehyde (MDA). Briefly, 1 mL of kidney homogenate was mixed with 2 mL of trichloroacetic acid–thiobarbituric acid−HCl reagent (15% trichloroacetic acid, 0.67% thiobarbituric acid, and 0.25 N HCl) and boiled at 100°C for 15 min. After cooling, the mixture was centrifuged at 3,000 rpm for 10 min. The supernatant was collected, and the absorbance was measured at 535 nm wavelength. MDA concentration was calculated using a molar absorption coefficient of 1.56 × 10^5^/M cm and expressed as nmol/mg protein [13].

### 2.4 Histopathological examination of renal tissue

4 μm paraffin-embedded sections were subjected to routine H&E staining for assessment of the histopathological changes of the kidney. The degree of tubular injury was scored according to the previously described method [11]. Briefly, under the light microscope (Leica, Wetzlar, Germany), at least ten fields in the cortex for each mouse were randomly selected to count the number of injured renal tubular including dilation, necrosis, and tubular formation by trained personnel who was blinded to the interventions. After then, a score was given to each mouse based on the percentage of damaged renal tubules to the total renal tubules: 0, less than 5%; 1, 5–25%; 2, 25–50%; 3, 50–75%; 4, over 75%.

### 2.5 Western blot analysis

Frozen renal cortex was lysed in the cell lysis buffer containing I and II inhibitor cocktails (Sigma, MO) for 20 min on ice. Samples were centrifuged twice, and the supernatants were obtained to measure the total protein concentration by Bradford’s method. The supernatant was then heated to 100°C with loading buffer for 5 min and separated on 8–15% SDS-PAGE gels and transferred onto PVDF membranes (Millipore, USA) following standard protocol. The PVDF membranes were incubated with primary antibodies against cleaved caspase-3 (1:1,000, Cell Signaling Technology, UK), Phospho-JNK (Thr183/Tyr185) (1:1,000, Cell Signaling Technology, UK), JNK (1:1,000, Cell Signaling Technology, UK), γH2AX (1:1,000, Abcam, USA), and GAPDH (1:1,000, Cell Signaling Technology, UK) overnight at 4°C. The membranes were washed by TBST buffer and incubated with secondary antibody for 1 h at room temperature. Target proteins were then visualized using an ECL Plus kit (Amersham, IL, USA) and analyzed using ImageJ software (National Institutes of Health, Bethesda, MD, USA).

### 2.6 Terminal deoxynucleotidyl transferase-mediated dUTP-biotin nick end labeling (TUNEL) assay

The paraffin-embedded kidney sections were exposed to the TUNEL reaction mixture (*in situ* cell death detection kit, POD) according to the manufacturer’s instructions (Roche Diagnostics, Basel, Switzerland) to detect the level of renal tubular epithelial cell apoptosis. The number of apoptotic cells in 10 fields per section and five sections per kidney was counted by identifying cells with TUNEL-positive nuclei under fluorescence microscopy.

### 2.7 Statistical analysis

Statistical analysis was performed using SPSS19.0 (SPSS, Inc., IL, USA). Data were expressed as mean ± SD and analyzed with independent samples *t*-test or one-way ANOVA. *P* < 0.05 was considered statistically significant.

## 3 Results

### 3.1 HHcy exacerbates IRI-induced renal dysfunction and oxidative stress

**Figure 1:**
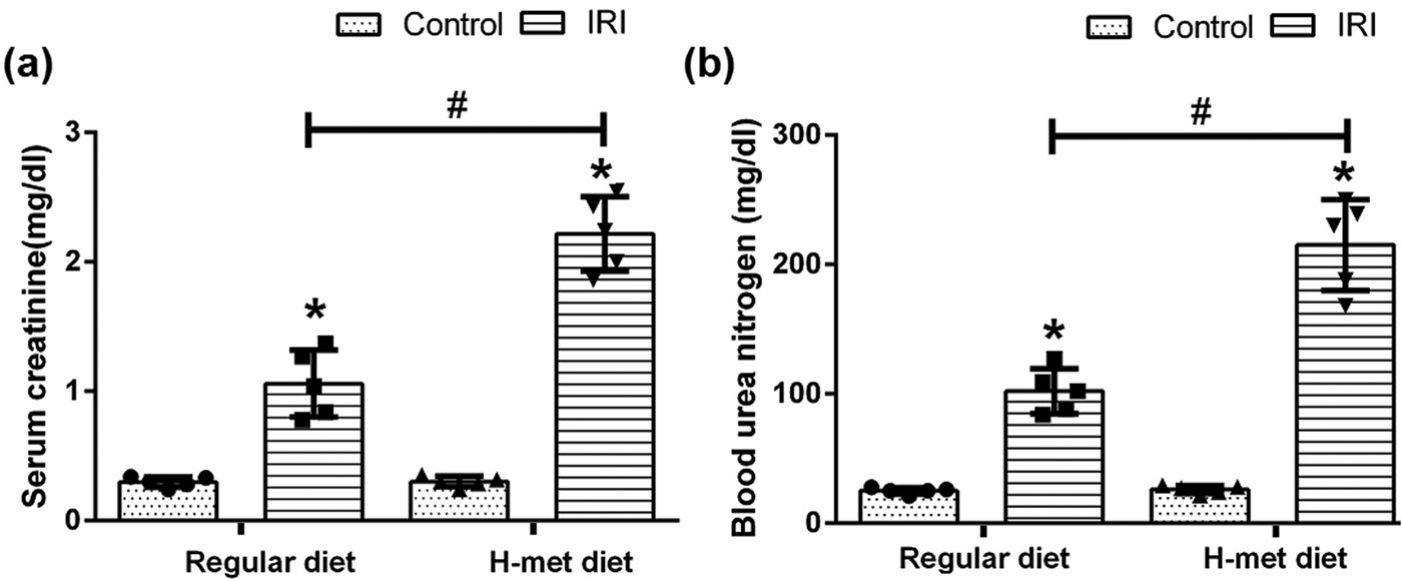
HHcy exacerbates IRI-induced renal dysfunction. (a) The level of serum creatinine in mice. (b) The level of blood urea nitrogen (BUN) in mice. Data are expressed as mean ± SD, *n* = 5. *P < 0.05 vs the control group under same diet; ^#^ *P* < 0.05 vs the IRI group pretreated with regular diet. H-met diet: High methionine diet.

After 2 weeks of diet treatment, the level of serum Hcy in the H-Met diet group was significantly higher than that of the regular diet group (35.01 ± 7.41 vs 9.15 ± 0.68, *P* < 0.05). Twenty four hour after reperfusion, serum creatinine and BUN levels were significantly higher in IRI mice than that of the control mice under both regular diet and H-Met diet pretreated conditions. Notably, compared with regular diet pretreatment, the H-Met diet didn’t change serum creatinine and BUN levels in control mice, but significantly increased both of them in IRI mice (Figure 1a and b).

**Figure 2:**
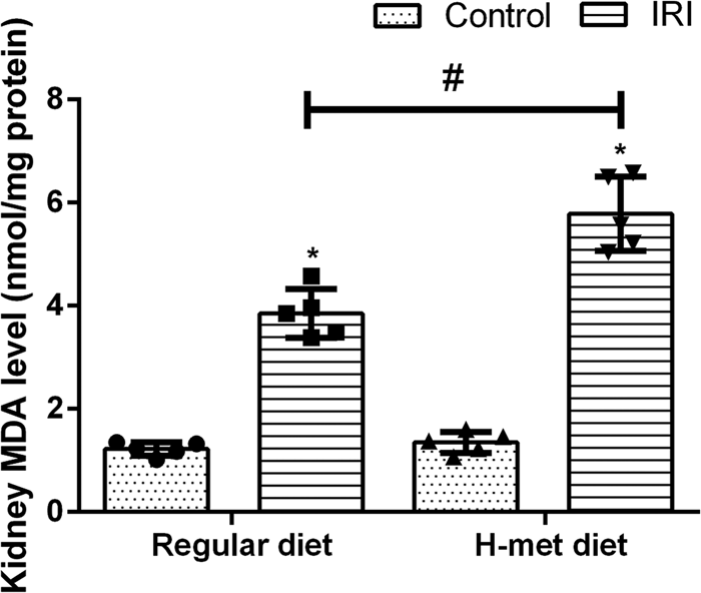
HHcy exacerbates IRI-induced oxidative stress. Data are expressed as mean ± SD, *n* = 5. *P < 0.05 vs the control group under same diet. ^#^ *P* < 0.05 vs the IRI group pretreated with regular diet. MDA: Malondialdehyde; H-met diet: High methionine diet.

To determine the influence of HHcy on oxidative stress, the level of MDA (an indicator of lipid peroxidation) was measured. As shown in Figure 2, the MDA level was significantly increased in IRI mice compared with the control mice; particularly, H-Met diet-pretreated IRI mice had the highest MDA level among mice. Our results indicate that preexisted HHcy condition can exacerbate IRI-induced renal dysfunction and oxidative stress.

### 3.2 HHcy exacerbates IRI-induced tubular injury and DNA damage

**Figure 3:**
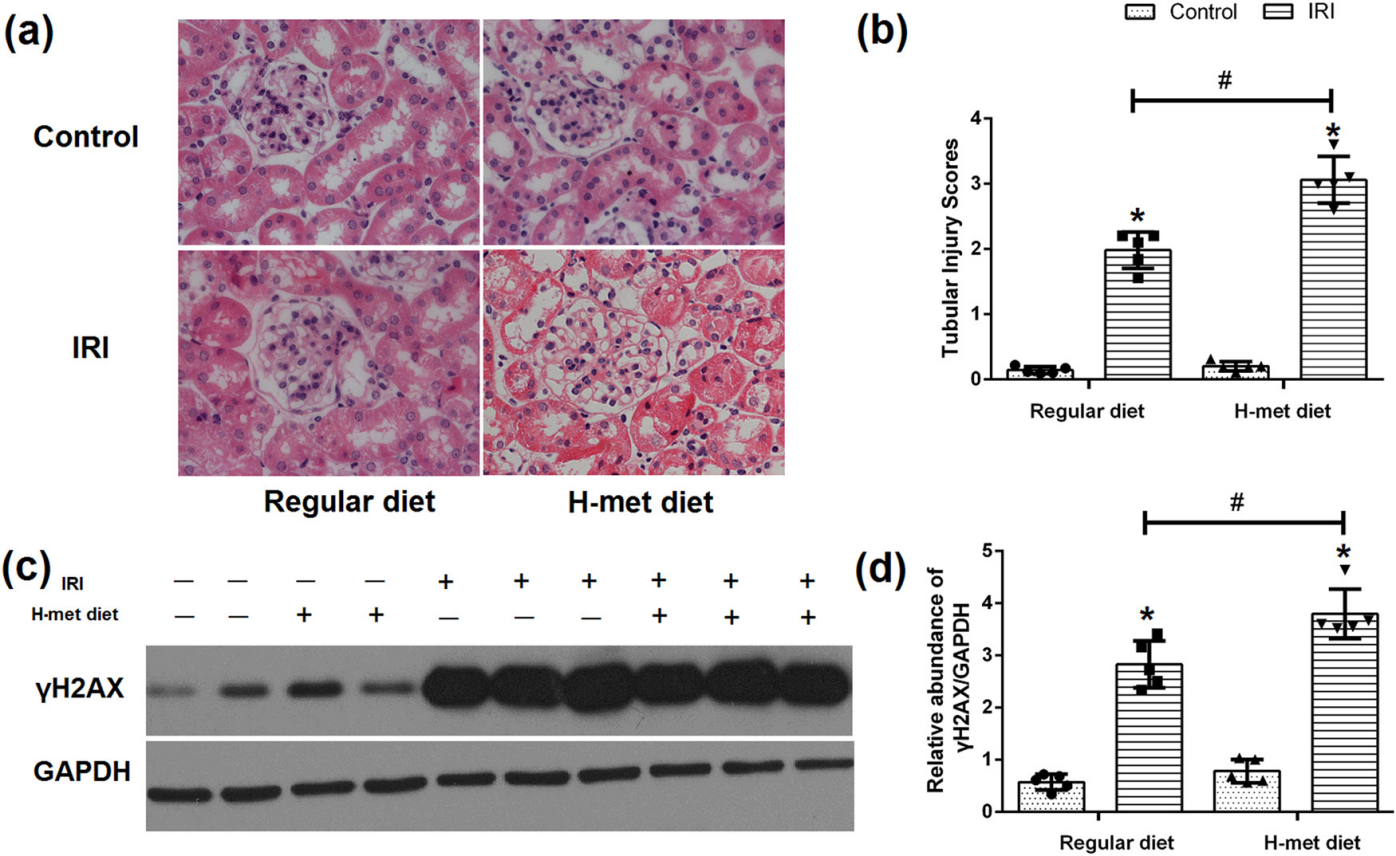
HHcy exacerbates IRI-induced tubular injury and DNA damage. (a) Representative figures of H&E staining show the tubular injury at 24 h after reperfusion. (b) Quantification assessment of tubular injury on the basis of H&E staining. (c) Representative western blot figures of γH2AX. (d) Graphic representation of relative expression of γH2AX normalized to GADPH. Data are expressed as mean ± SD, *n* = 5. *P < 0.05 vs the control group under same diet. ^#^ *P* < 0.05 vs the IRI group pretreated with regular diet. H-met diet: High methionine diet.

Twenty four hours after reperfusion, tubular injuries such as necrosis, dilatation, and cell swelling were observed in IRI mice under both regular diet and H-Met diet pretreated conditions. Notably, the degree of renal tubular injury was significantly severer in the H-Met diet pretreated IRI mice than regular diet pretreated IRI mice (Figure 3a and b), suggesting that preexisted HHcy condition can exacerbate IRI-induced tubular injury.

To determine the HHcy’s influence on the DNA damage, western blot analysis was performed to confirm the expression of γH2AX. As shown in Figure 3c and d, γH2AX was significantly increased in IRI mice, especially in H-Met diet pretreated IRI mice, indicating that preexisted HHcy condition can exacerbate IRI-induced DNA damage.

### 3.3 HHcy promotes the activation of JNK pathway in IRI-induced AKI mice

**Figure 4:**
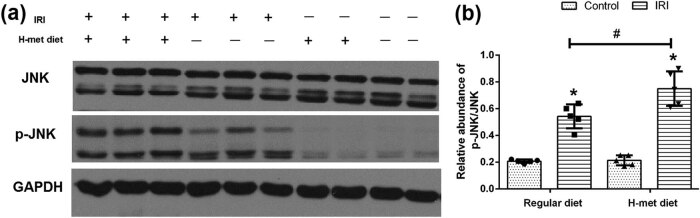
HHcy promotes IRI-induced JNK pathway activation. (a) Representative western blot figures of JNK and p-JNK. (b) Graphic representation of the ratio of p-JNK/JNK. Data are expressed as mean ± SD, *n* = 5. *P < 0.05 vs the control group under same diet. ^#^ *P* < 0.05 vs the IRI group pretreated with regular diet. H-met diet: High methionine diet.

To determine the potential influence of HHcy on the JNK pathway, western blot analysis was performed to examine the expression of JNK and p-JNK. As shown in Figure 4a and b, JNK pathway activation manifested as increased expression of p-JNK was noticed in IRI mice, particularly in H-Met diet pretreated IRI mice. Our results suggest that preexisted HHcy condition can promote the activation of the JNK pathway caused by IRI.

### 3.4 HHcy exacerbates IRI-induced renal tubular epithelial cell apoptosis

**Figure 5:**
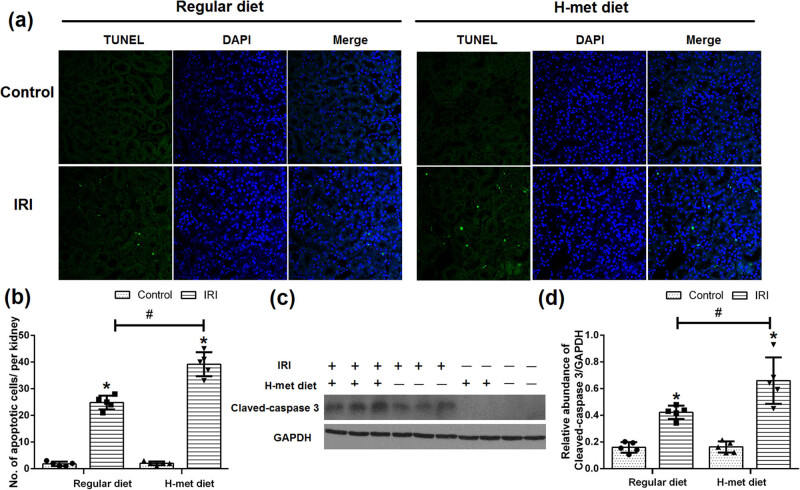
HHcy exacerbates IRI-induced renal tubular epithelial cell apoptosis. (a) Representative immunofluorescence figures show apoptotic cell death detected by TUNEL staining at 24 h after reperfusion. (b) Quantitative determination of apoptotic tubular cells. The number of apoptotic cells was counted in 10 fields per section and five sections per kidney. (c) Representative western blot figures of cleaved caspase-3. (d) Graphic representation of cleaved caspase-3 normalized to GADPH. Data are expressed as the mean ± SD, *n* = 5. *P < 0.05 vs the control group under same diet. ^#^ *P* < 0.05 vs the IRI group pretreated with regular diet. H-met diet: High methionine diet.

To determine the potential effect of HHcy on the renal tubular epithelial cell apoptosis, TUNEL assay was performed. As shown in Figure 5a and b, only very few apoptotic cells were detected in control animals under both regular diet and H-Met diet pretreated conditions, while more tubular apoptotic cells were noticed in the H-Met diet pretreated IRI mice than regular diet pretreated IRI mice. Determination of cleaved caspase-3 by western blotting further confirmed that severer apoptosis was existed in the H-Met diet pretreated IRI mice (Figure 5c and d). Our results suggest that preexisted HHcy condition can exacerbate IRI-induced renal tubular epithelial cell apoptosis.

## 4 Discussion

In the present study, we used high methionine diet to generate HHcy mice; after that, the IRI-induced AKI model was employed to explore the effect and potential mechanisms of HHcy on IRI-induced AKI. Our results showed that the preexisted HHcy condition exerts very little influence on normal mice, but significantly exacerbates the renal damage, characterized by a decline in renal function and increase of tubular injury of IRI-induced AKI mice, which may be related to its potential in mediating oxidative stress, DNA damage, JNK pathway, and apoptosis.

In humans, two metabolism pathways, including remethylation and transsulfuration, are mainly involved in the metabolism of Hcy. It has been reported that up to 70% of plasma Hcy is removed from the kidney, mainly through transsulfuration [14]. Therefore, kidney injury can increase plasma Hcy concentration; in return, increased Hcy may be harmful to the kidney. In a study performed by Ye et al., the authors found that CKD patients with HHcy had higher incidence of renal damage than patients with normohomocysteinemia [15]. Liu and colleagues retrospectively analyzed 7,240 hypertensive patients and found that patients who developed HHcy had a higher long-term rate of renal function decline compared with patients who didn’t develop HHcy [16]. In an animal study, authors found renal dysfunction appeared in cystathionine β-synthase-deficient HHcy mice compared with wild-type mice [17]. In the present study, we noticed that HHcy almost doesn’t influence the renal function and tubular structure of control mice, which was consistent with the observations of Li et al. [18]. We speculate that the detrimental role of Hcy may not appear because of the moderate concentration and short action time of Hcy. While, on the other hand, our results showed that HHcy could exacerbate the renal damage of IRI-induced AKI mice.

Renal tubular epithelial cells account for about 70% of renal parenchymal cells in the kidney and are vulnerable to ischemia, hypoxia, nephrotoxin, and immune inflammation during acute renal injury because of their nature of high oxygen consumption. In the present study, significant renal damage, including tubular epithelial cell apoptosis and necrosis, was noticed in the IRI-induced AKI mice, consistent with previous studies [19,20]. Notably, the renal damage was severer in the H-Met diet pretreated IRI mice than regular diet pretreated IRI mice. To determine the potential mechanisms of this phenomenon, we further focused on the changes of oxidative stress, DNA damage, and the JNK pathway because they are closely related to tubular epithelial cell apoptosis.

As we know, oxidative stress is a well-known hallmark of IRI-induced AKI, which is believed to be one of the critical factors causing kidney injury during ischemia-reperfusion [21]. DNA damage, a deleterious event that occurs in the genome, may be induced under many conditions such as oxidative stress or free radical insult, irradiation, and UV exposure [22]. Increasing evidence indicated that oxidative stress and DNA damage occur in kidney tissues following ischemia-reperfusion [13,20]. In response to DNA damage, several pathways such as NF-κB and JNK pathways may be activated to maintain genome homeostasis [23]. Besides, endoplasmic reticulum stress, induced by oxidative stress, hypoxia, or energy deprivation, also existed in IRI-induced AKI, which can trigger the activation of the JNK pathway [5]. In the present study, our results showed that the expression of MDA, γH2AX, and p-JNK, as well as the number of apoptotic tubular epithelial cells, was significantly higher in the H-Met diet pretreated IRI mice than regular diet pretreated IRI mice. We speculate that HHcy might exhibit its detrimental role in IRI-induced AKI mice by promoting oxidative stress, which causes DNA damage and triggers apoptosis through activation of the JNK pathway. Considering the detrimental role of HHcy, close attention should be paid to it in patients vulnerable to IRI-induced AKI.

However, this study has limitations. The most noticeable one is that this *in vivo* study is not sufficient to make a definite conclusion. In the future, activation or inhibition experiments focused on oxidative stress, DNA damage, or JNK pathway are warranted to further explore the relationship between HHcy and IRI-induced AKI.

## 5 Conclusion

Our results demonstrated that preexisted HHcy condition could exacerbate IRI-induced AKI, which may be achieved through promoting oxidative stress, DNA damage, JNK pathway activation, and consequent apoptosis.
